# Supplementary material for: MetaRibo-Seq measures translation in microbiomes
Source: Nat Commun. 2020 Jun 29;11:3268. doi: 10.1038/s41467-020-17081-z (PMC7324362; doi:10.1038/s41467-020-17081-z)
Supplement: Supplementary file 10 — Supplementary Data 7 [file 41467_2020_17081_MOESM10_ESM.zip › File2/Confidence_High_Taxonomy/209478_out.krona.html]

Javascript must be enabled to view this page.

members
magnitude
magnitudeUnassigned
count
unassigned
taxon
rank

209478\_out

3

3
2
superkingdom

phylum
1224
1

1
28216
class

order
1
80840

1
80864
family

1
80865
genus

species

SRS1054691\_contig\_number\_contig-100\_1458.39491
1
80866

201174
1
phylum

1
1760

SRS048060\_contig\_number\_contig-100\_14386.14387
class

phylum
1239
1

186801
1
class

order
186802
1

1
541000
family

1627893
1
species

SRS148196\_contig\_number\_contig-100\_2703.235205
